# Editorial: Exploring genetic characteristics and molecular mechanisms of host adaptation of viruses with artificial intelligence (AI) or (and) biological (BIO) approaches

**DOI:** 10.3389/fcimb.2024.1474097

**Published:** 2024-08-26

**Authors:** Jing Li, Xiao-He Li, Esmaeil Ebrahimie, Lei Huang

**Affiliations:** ^1^ State Key Laboratory of Pathogen and Biosecurity, Academy of Military Medical Science, Beijing, China; ^2^ College of Basic Medical Sciences, Inner Mongolia Medical University, Hohhot, China; ^3^ Genomics Research Platform, School of Agriculture, Biomedicine and Environment, La Trobe University, Melbourne, VIC, Australia; ^4^ School of Animal and Veterinary Science, The University of Adelaide, Adelaide, SA, Australia; ^5^ School of BioSciences, The University of Melbourne, Melbourne, VIC, Australia; ^6^ Senior Department of Infectious Diseases, The Fifth Medical Center of Chinese PLA General Hospital, Beijing, China

**Keywords:** host adaptation, genetic characteristics, molecular mechanisms, virus, artificial intelligence, biological approach

Most types of viruses cause infection and transmission in a limited range of hosts, indicating viral species tropism or host adaptation. Such host adaptation by viruses manifests as various genetic characteristics-associated molecular mechanisms in both the virus and host. Viral glycoprotein cleavage by host protease repertoire exerts a role in the zoonotic potential and risk posed by influenza A virus and coronavirus (Heindl and Bottcher-Friebertshauser, 2023). Interactions between viral polymerase PB2 subunit and host ANP32A (Camacho-Zarco et al., 2020), between polymerase units (Li et al., 2011), or even between the untranslated region of polymerase gene and the polymerase (Sun et al., 2014) are also involved in viral host adaptation. Artificial intelligence (AI)-based approaches have revealed the importance of viral genomic dinucleotides or dinucleotide clusters (Li et al., 2022, 2020) or mutations in viral proteins (Nan et al., 2022; Serna et al., 2022) to the host adaptation of severe acute respiratory syndrome coronavirus 2 (SARS-CoV-2). In light of the high performance by attention mechanism to capture the contextual words or sentences in natural language processing (NLP) (Vaswani et al., 2017), more NLP and other AI approaches should promise to swiftly and accurately facilitate the identification adaptive mutations with epistatic/hypostatic interactions or other types of significant association. And such genomic contexts or mutations important to adaptation have also been validated by experimental biology (BIO) approaches (Bugatti et al., 2023; He et al., 2022; Lista et al., 2022; Mishra et al., 2022; Supasa et al., 2021). Particularly, high-throughput methods such as deep mutational scanning have identified highly important mutations underlying the molecular mechanisms of host adaptation of SARS-CoV-2 (Dadonaite et al., 2024; Frank et al., 2022; Starr et al., 2020). Several molecular mechanisms by which other viruses adapt to the host have been recognized by AI and/or BIO approaches. An in-depth mutational analysis has revealed the potential role of host APOBEC3 in human adaptation of monkeypox virus (MPXV) in ongoing microevolution (Isidro et al., 2022). GP-A82V mutation-mediated increase in infectivity was found to correlate with disease severity during the Ebola virus disease epidemic, suggesting that this mutation was related to the adaptation of the virus to the human host (Diehl et al., 2016). However, previous studies or methodologies with multiple biological or virological methods was time-consuming, sporadic, and lacked predictability. Moreover, the accumulated huge amount of BIO results has not been deep learned to uncover the molecular mechanism in more details underlining viral adaptation to host. AI approaches for exploring genetic characteristics and its associated molecular mechanisms underlying host adaptation of viruses would be beneficial.

Recent studies have explored the genome-adaptation association using AI and/or BIO approaches. A cohort study by Silva et al. (Analysis of associations between the TLR3 SNPs rs3775291 and rs3775290 and COVID-19 in a cohort of professionals of Belém-PA, Brazil) revealed an association of single nucleotide polymorphisms (rs3775291 and rs3775290) in toll-like receptor 3 (TLR3) with COVID-19 severity in a cohort of professionals in Belém-PA, Brazil, implying host restriction to the SARS-CoV-2 adaptation to humans. The involvement of TLR3 has also been implied in the SARS-CoV-2-induced senescence in human cells (Tripathi et al., 2021). A more complicated virus-host co-evolution or viral adaptation to host was indicated in a study by Wang et al. (Comprehensive characterization of ERV-K (HML-8) in the chimpanzee genome revealed less genomic activity than humans); the study comprehensively characterized an endogenous retrovirus (HML-8), which originate from ancestral germline infection, in chimpanzee and humans, revealing less activity of the chimpanzee genome compared to that of the human genome.

Meanwhile, adaptive viral genomes have been recognized in various types of viruses. A study by Rashid et al. (Characterization of HIV-1 CRF02_AG/A3/G unique recombinant forms identified among children in Larkana, Pakistan) indicated that highly mutated retroviruses of human immunodeficiency virus (HIV) recombined with each other to adapt to the host. The rapid mutation and adaptation of HIVs has been associated with molecular mechanisms such as the adaptation to human leukocyte antigen-associated immune pressures (Avila-Rios et al., 2019; Carlson et al., 2015; Kloverpris et al., 2015) or to specific immunity (Da, 2003). The host adaptation of DNA viruses has also been revealed, particularly with AI approaches. A linear adaptation of MPXV has been indicated by genomic composition-based machine learning approach (Zhang et al., 2024). Genome composition-based deep learning approaches have also predicted the oncogenic potential of human papillomaviruses in a study by Hao et al. (Genome composition-based deep learning predicts oncogenic potential of HPVs). The virus-host adaptation has also been recognizable in bacteriophages. A spontaneous tail tubular mutation was indicated to drive the host range expansion of Acinetobacter phage vB_Ab4_Hep4 in a study by He et al. (Host range expansion of Acinetobacter phage vB_Ab4_Hep4 driven by a spontaneous tail tubular mutation). Such kind of adaptation has also been validated experimentally through strong parallel adaptation, a repeated and independent evolution of similar genotypes/traits from a common ancestor (Burmeister et al., 2023).

Taken together, although virus-host co-evolution or viral adaptation to host has been uncovered in several studies, it is far from being recognized systematically, particularly, in the context of an association between genetic characteristics and molecular phenotypes (mechanisms). Progress in this area has been slow owing to the high complexity of virus-host interactions, which is difficult to elucidate with traditional analysis or experiments. However, this methodological bottleneck is expected to be overcome using AI and high-throughput BIO approaches. Therefore, it will be exciting to see how these approaches will propel this research field in the future by uncovering the genetic characteristics and molecular mechanisms of host adaptation of viruses.
